# Biological Oxidation of Manganese Mediated by the Fungus *Neoroussoella solani* MnF107

**DOI:** 10.3390/ijms242317093

**Published:** 2023-12-04

**Authors:** Shiping Wei, Wenxiu Wang, Feirong Xiao

**Affiliations:** 1 Key Laboratory of Polar Geology and Marine Mineral Resources (China University of Geosciences, Beijing), Ministry of Education, Beijing 100083, China; 2 School of Marine Sciences, China University of Geosciences, Beijing 100083, China; wangwx@sustech.edu.cn (W.W.); xfeirong@163.com (F.X.)

**Keywords:** manganese-oxidizing fungus, manganese oxides, birnessite, ramsdellite, *Neoroussoella solani*

## Abstract

Manganese oxides are highly reactive minerals and influence the geochemical cycling of carbon, nutrients, and numerous metals in natural environments. Natural Mn oxides are believed to be dominantly formed by biotic processes. A marine Mn-oxidizing fungus *Neoroussoella solani* MnF107 was isolated and characterized in this study. SEM observations show that the Mn oxides are formed on the fungal hyphal surfaces and parts of the hypha are enveloped by Mn oxides. TEM observations show that the Mn oxides have a filamentous morphology and are formed in a matrix of EPS enveloping the fungal cell wall. Mineral phase analysis of the fungal Mn oxides by XRD indicates that it is poorly crystalline. Chemical oxidation state analysis of the fungal Mn oxides confirms that it is predominantly composed of Mn(IV), indicating that Mn(II) has been oxidized to Mn (IV) by the fungus.

## 1. Introduction

Manganese oxides are widespread in aquatic and terrestrial environments [1,2] and have various forms, such as fine aggregates, veins, nodules, concretions, crusts, dendrites, and coatings on other mineral and rock surfaces [3]. Manganese oxides can serve as excellent electron acceptors during the oxidation of organic matter [4,5,6,7] and possess high adsorption capacity for toxic trace metals (e.g., Cu, Co, Cd, Zn, Ni, Pb, Fe, U, As, and Se) [8,9,10,11,12,13,14]. In addition, manganese oxides play an important role in preserving organic matter, affect the nutrient bioavailability of N, P and K [15,16,17,18], and may affect the migration, transformation, and fate of sulfur [19,20]. Therefore, manganese oxides are thought to play a crucial role in regulating the distribution of the biogeochemical cycling of C, N, S, Mn, Fe, and trace metals [10,21,22]. 

Manganese is distributed widely throughout the natural environment, taking the three dominant valence states of Mn(II), Mn(III), and Mn(IV). The Mn cycles among those different states are driven either by chemical or microbial processes. Mn (II) can be oxidized to Mn (III) or further to Mn (IV) in natural environments, but the reaction rate is very slow [21]. Comparatively, the rate of microbial oxidation of Mn(II) can exceed that of abiotic oxidation by up to five orders of magnitude [10,23]. Thus, natural Mn oxides are believed to be mainly formed by biological processes [24]. Earlier research has demonstrated that, aside from bacteria capable of oxidizing manganese [21,25,26,27,28,29,30], certain fungi also exhibit the ability to oxidize manganese, including *Acremonium strictum*, *Alternaria alternata*, *Coniothyrium fuckelli*, *Cladosporium cladosporioides*, *Phoma glomerata*, *Paraconiothyrium sporulosum*, *Periconia* sp., *Plectosphaerella cucumerina*, *Pyrenochaeta* sp., *Stagonospora* sp., *Stilbella aciculosa*, and *Phanerochaete chrysosporium* [1,31,32,33,34,35,36,37,38,39,40,41]. Phylogenetic analysis reveals that these fungi belong to the phylum *Ascomycota* or *Basidiomycota*. Mn-oxidizing fungi have faster manganese oxidation rates and higher tolerance to dissolved manganese than manganese-oxidizing bacteria [41,42]. It is believed that fungi play critical roles in manganese oxidation in the Earth’s surface environments [9].

In this study, we characterized the manganese oxides formed by an *Ascomycota* fungus, *Neoroussoella solani* MnF107, which was isolated from marine sediment. The results provide a better understanding of the characteristics and mechanisms of fungal manganese oxidization and lay a foundation to investigate their oxidization mechanisms and ecological roles in marine environments.

## 2. Results

### 2.1. Isolation and Identification of the Mn(II)-Oxidizing Fungus

**Figure 1:**
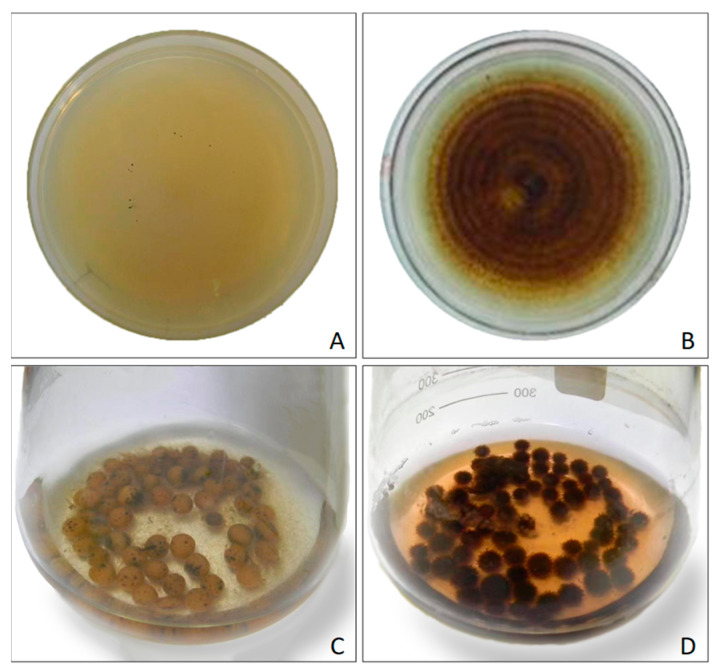
Morphologies of the fungal strain MnF107 growing on liquid (**A**,**B**) and solid media (**C**,**D**). (**A**,**C**), without Mn^2+^; (**B**,**D**), with Mn^2+^.

The fungus strain designated MnF107 was isolated and confirmed as a Mn(II)-oxidizing fungus. Manganese oxidization by MnF107 occurred in both solid and liquid media containing 1 mM Mn^2+^ (Figure 1A–D). When it grew on the solid medium without Mn^2+^ (Figure 1A), the fungal hyphae were pale white and grew radially outward from the central inoculation point. In contrast, the fungal hyphae presented a dark brown color on the medium with Mn^2+^ (Figure 1B), indicating soluble Mn(II) was oxidized to solid Mn oxides. A subsequent observation of its growth in the liquid medium was performed. In the liquid AY medium without Mn^2+^, the growing fungus formed pale white mycelial balls (Figure 1C), whereas dark brown mycelial balls appeared in the presence of Mn^2+^ medium (Figure 1D), suggesting manganese oxidation occurred close to the fungal hyphal surfaces. 

**Figure 2:**
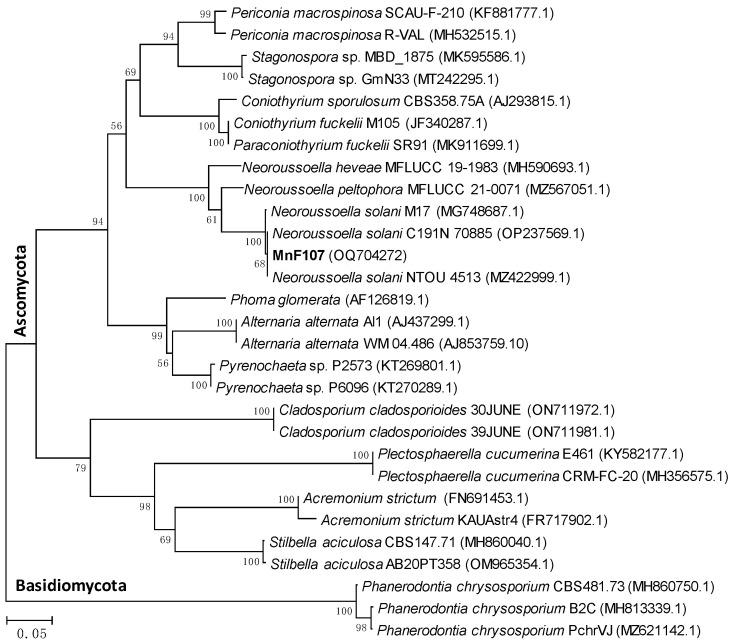
Neighbor-joining tree showing the phylogenetic position of strain MnF107 and related species of Mn-oxidizing fungi based on ITS sequences. Bootstrap values are based on 1000 replicates and are shown at the nodes. GenBank accession numbers are given in parentheses. The scale bar represents a 1% nucleotide sequence divergence.

The ITS sequence of strain MnF107 showed 99.5% identity to *Neoroussoella solani* C191N (accession number: OP237469.1). Phylogenetic analysis indicated that strain MnF107 belonged to the genus *Neoroussoella* (Figure 2), which is affiliated with the phylum *Ascomycota*. The ITS sequence of strain MnF107 has been deposited in the GenBank database under the accession number OQ704272.

### 2.2. SEM, EDS, and TEM Characterizations of the Manganese Oxides

**Figure 3:**
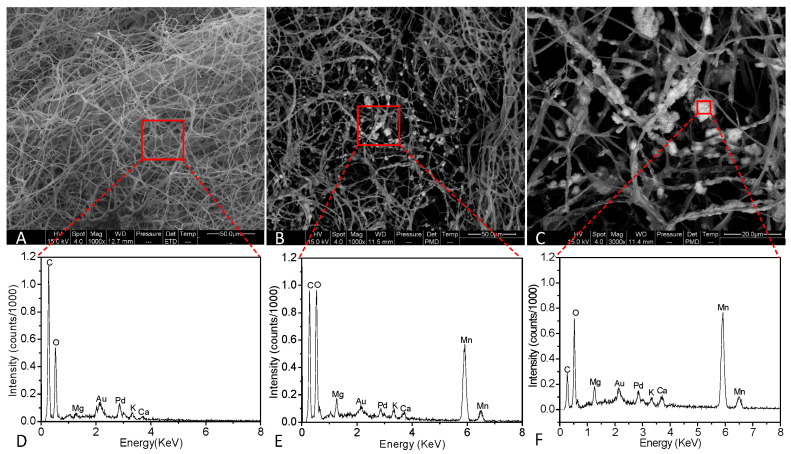
SEM images (**A**–**C**) and EDS spectra (**D**–**F**) of the manganese oxides formed by MnF107. (**A**), the fungal hypha grown in AY medium without Mn^2+^; (**B**,**C**), the fungal hypha grown in AY medium supplemented with 1 mM Mn^2+^. The red squares show regions used for EDS spectra analysis.

SEM images of the fungal Mn oxides associated with the fungal hyphae are shown in Figure 3A–C. This fungal strain displays filamentous mycelia without Mn oxide particles on the hyphal surface when incubated in AY medium without Mn^2+^ (Figure 3A). However, many Mn oxide particles were observed to coat on the hyphal surface (Figure 3B,C) when AY liquid medium was supplemented with Mn^2+^. With time, parts of the mycelium were gradually enveloped by Mn oxides (Figure 3C), indicating Mn(II) oxidation first occurs around the fungal cell wall.

The EDS spectra of the elemental composition of MnF107 grown in AY media with or without Mn^2+^ show a marked difference (Figure 3D–F). The culture from the medium without Mn^2+^ shows high carbon and oxygen contents from fungal mycelia (Figure 3D). The ratio of carbon to oxygen (C:O) was relatively higher in the medium without Mn^2+^ than with Mn^2+^ (Figure 3D,E). More importantly, the manganese content was higher in the culture with Mn^2+^ (Figure 3E). A close examination of the aggregated fungal deposits showed it was mainly composed of manganese and oxygen (Figure 3F).

**Figure 4:**
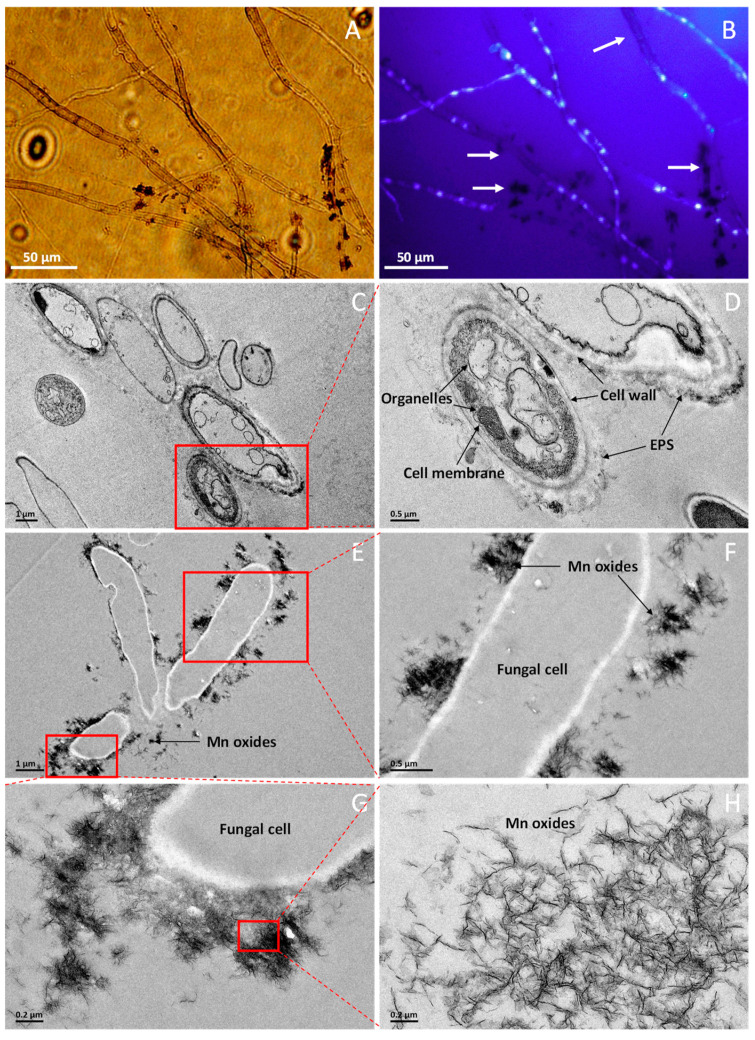
Light, fluorescence, and transmission electron (TEM) microscopic characterizations of fungal cells and manganese oxides produced by MnF107. (**A**,**B**) light and fluorescence microscopic comparison of fungal hyphae growing on cellophane membranes supported by solid media with DAPI; (**C**,**D**) cross-section through the fungal hypha grown in AY medium without Mn^2+^; (**E**–**G**) cross-section through the fungal hypha grown in AY medium supplemented with 1 mM Mn^2+^; (**H**) features of Mn oxides on the fungal cell surface.

Comparative observations of the hyphae grown on media supplemented with DAPI under light (Figure 4A) and fluorescence microscopes (Figure 4B) showed that the fungal nuclear staining with DAPI resulted in no fluorescence in the positions of manganese oxide deposition (the arrows in Figure 4B). In contrast, the fungal cells without manganese oxide deposition on their surface fluoresced brightly (Figure 4B). This finding suggests that the fungal cells with Mn oxides deposited on its surfaces exhibit probable non-viable or fluorescence quenching caused by manganese irons. 

Comparative TEM observations of the hyphae grown in AY media supplemented with and without Mn^2+^ were performed (Figure 4C–H). The TEM examinations showed that well-defined cell walls, cell membranes, and organelles were observed for the fungal hypha grown on medium without Mn^2+^ (Figure 4C,D). However, the intracellular organelles were not observed for the fungal cells grown on medium with 1 mM Mn^2+^ (Figure 4E,F), which may be due to the thin-sectioning of the fungal cells, which limits the observation of the organelles. In addition, the TEM images show that the location and morphology of fungal Mn oxides are associated with the fungal cells (Figure 4E–G). A layer of extracellular polymeric substances (EPS) can be clearly observed surrounding the fungal cell wall (Figure 4C–G), and the Mn oxides appear to aggregate on the fungal hyphal surfaces or adjacent to the fungal cell wall (Figure 4E–G). A closer examination of the fungal Mn oxides revealed that a filamentous structure (Figure 4H) is a typical morphology of fungus-formed Mn oxides [1,43].

### 2.3. Composition and Structure Characterizations of Fungal Mn Oxides

**Figure 5:**
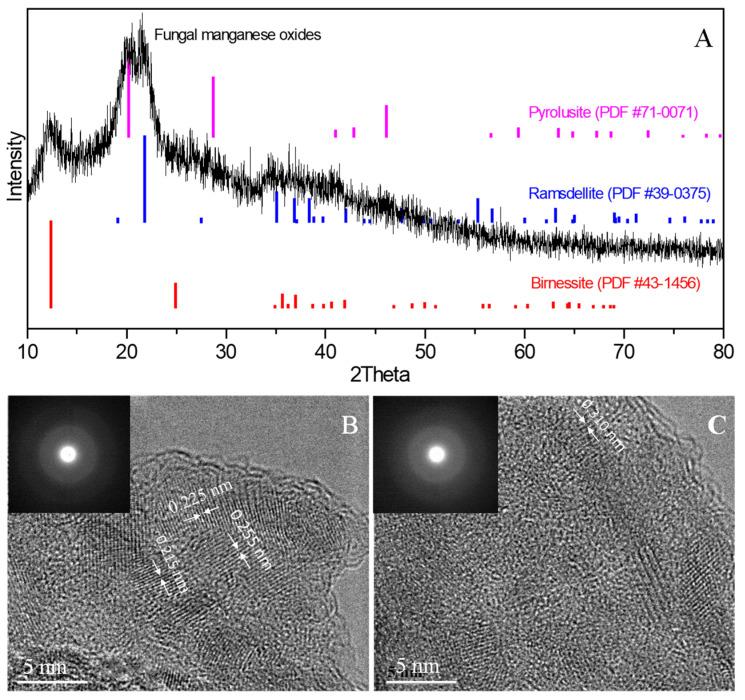
XRD diffraction patterns (**A**) and HRTEM characterizations (**B**,**C**) of fungal manganese oxides formed by MnF107. Insets in (**B**,**C**) show the SAED patterns of manganese oxides.

XRD spectra of the fungal Mn oxides show a noisy background with three broad peaks, indicating that they are poorly crystalline (Figure 5A). The poor crystallinity of these minerals makes them difficult to accurately identify, and the peaks at 2θ of 12.4°, 20.2°, and 21.8° might be assigned to birnessite (δ-MnO_2_), pyrolusite (β-MnO_2_), and ramsdellite (γ-MnO_2_), respectively [3,44]. A broad peak at 2θ from 35° to 42° might be tentatively assigned to birnessite and ramsdellite. HRTEM and SAED were carried out to confirm the results of XRD. The measured d-spacing values of 0.215 nm and 0.225 nm correspond to the (1 1 $\bar{2}$) plane and (1 1 1) plane of birnessite; that of 0.255 nm corresponds to the (3 0 1) plane of ramsdellite (Figure 5B), and that of 0.310 nm corresponds to the (1 1 0) plane of pyrolusite (Figure 5C). However, selected area electron diffraction (SAED) cannot unequivocally distinguish the possible MnO_2_ polymorphs as they show diffuse rings, indicating the fungal MnO_2_ are poorly crystalline (insets of Figure 5B,C).

**Figure 6:**
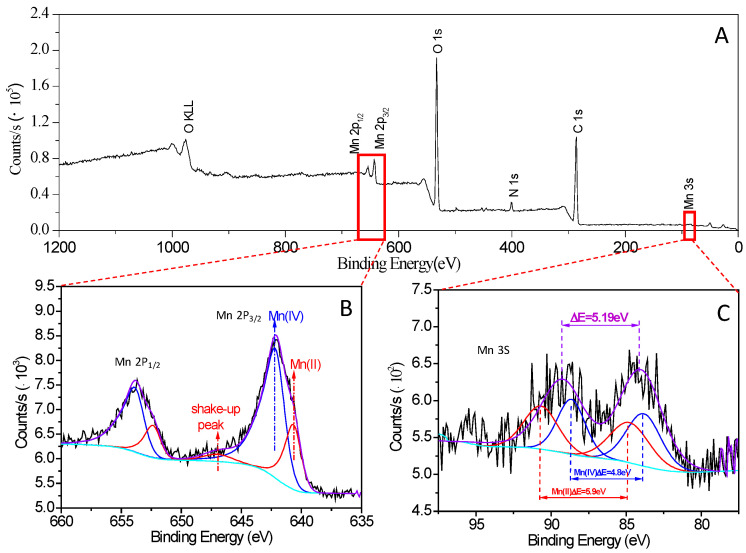
XPS spectra of fungal manganese oxides. (**A**), survey scan; (**B**), Mn 2p region; (**C**), Mn 3s region. The black curve represents the spectral data, and the purple curve represents the spectral fit using Mn^2+^ and Mn^4+^ multiple peaks. The red and blue curves are Mn^2+^ and Mn^4+^ multiple peaks, respectively. The Shirley background is shown as the curve.

XPS was used to identify the oxidation state of Mn in the fungal Mn oxides. The elemental signals of C, O, N, and Mn were observed in the XPS spectrum (Figure 6A), indicating the presence of Mn oxides in a fungal mycelial matrix. Figure 6B shows the XPS Mn 2p spectrum of fungal Mn oxides, where the Mn 2p regions consist of a spin-orbit doublet with Mn 2p_1/2_ and Mn 2p_3/2_. The asymmetric Mn 2p_3/2_ main metal peak has a binding energy at 642.2 eV, with a splitting energy of 11.7 eV between Mn 2p_1/2_ and Mn 2p_3/2_, attributed to Mn(IV) in the fungal Mn oxides. Conversely, the Mn 2p_1/2_ and Mn 2p_3/2_ peaks at 652.4 eV and 640.7 eV, respectively, combined with a distinct shake-up peak (satellite peak) at 647 eV (Figure 6B), indicate the presence of Mn(II) in the fungal Mn oxides [45,46]. The atomic ratio of Mn(IV) and Mn(II) in the fungal Mn oxides is 67.7:32.3. Moreover, Mn 3s core-level spectra were also collected to confirm the oxidation state of manganese. As shown in Figure 6C, the Mn 3s core-level peaks (89.38 eV and 84.19 eV) show that the peak splitting energy is 5.19 eV, which falls between 4.8 eV and 5.9 eV, the mean multiplet splitting values for Mn(IV) and Mn(II). This result demonstrates the predominance of Mn(IV) in the fungal oxides due to fungal Mn(II) oxidization. The presence of Mn(II) may be the incompletely oxidized Mn(II), absorbed in the fungal oxides.

## 3. Discussion

Previous studies have shown that several fungi capable of oxidizing Mn(II) were isolated from various environments, such as soils [31,41], building stones [33], pebbles from stream water [35], acid mine drainage treatment systems [1,40], sediment from Mn-rich aquatic environments [37], and Mn nodules from rice fields [38]. The here-studied Mn-oxidizing fungus, *Neoroussoella solani* MnF107, isolated from a marine environment, belongs to the phylum *Ascomycete* (Figure 2). Previous studies have shown that the Mn-oxidizing fungi identified to date belong to the phylum *Ascomycete* or *Basidiomycota* [31,32,33,34,35,36,37,38,40,41], which were proved to have different Mn(II) oxidization mechanisms [40]. Mn(II) oxidization by a white-rot *Basidiomycete* such as *Phanerochaete chrysosporium* has been shown to be directly linked to lignocellulose degradation [47], and the Mn(II)-oxidizing *Ascomycetes* may have cellulose oxidization capacity during the Mn(II) oxidization processes [40]. *Neoroussoella* sp., formerly known as *Roussolella* sp., was detected in freshwater and marine environments [48,49]. It is known that Mn-oxidizing fungi play significant roles in the biogeochemical cycling of manganese in natural environments [21] and also have a huge application potential in environmental bioremediation to remove manganese and other heavy metals [42,50,51]. Further detailed studies need to be performed to shed light on *Neoroussoella solani* MnF107’s ecological roles in Mn cycling in the future.

Characteristics of Mn(II) oxidation by *Neoroussoella solani* MnF107 were observed by SEM, which shows that Mn(II) oxidation occurred initially in distinct locations along the hyphal surface (Figure 3B). Over time, parts of the mycelium were gradually enveloped by Mn oxides (Figure 3C), which is frequently observed in other manganese-oxidizing fungi, such as *Acremonium* sp. KR21-2, *Plectosphaerella cucumerina* DS2psM2a2, and *Cladosporium halotolerans* XM01 [1,35,36,41]. Previous studies have shown that laccase and peroxidase produced by fungi participate in the oxidation of Mn(II) [32,35]. In vitro experiments show that a band of laccase exercised from a gel of proteins is capable of depositing Mn oxides [35,36], suggesting that manganese oxidation requires that the enzyme contact the substrate directly. Because the Mn oxides are deposited on the fungal hyphal surface, it was inferred that enzymes associated with the cell wall are likely involved in manganese oxidation processes [1]. TEM observations showed that the Mn oxides were formed in the fungal EPS (extracellular polymeric substances) matrices surrounding the fungal hyphae. This is consistent with the observation by Emerson et al. [52] that the *Metallogenium*-like Mn oxides formed by a fungus were formed in a matrix of anionic polymers likely containing acid polysaccharides. 

The Mn oxides produced by *Neoroussoella solani* MnF107 accumulate as particles on the surface of fungal hyphae (Figure 3). TEM observation showed that these Mn oxide particles actually have a densely aggregated filamentous structure (Figure 4), which is similar to that of Mn oxides formed by Acremonium sp. KR21-1 [43]. Santelli et al. [1] observed four Mn oxidizing fungi, *Plectosphaerella cucumerina* DS2psM2a2, *Pyrenochaeta* sp. DS3Say3a, *Stagonospora* sp. SRC11sM3a, and *Acremonium strictum* DS1bioAY4a, and found that morphologies of the Mn oxides vary with these fungal species. Mn oxides produced by *Plectosphaerella cucumerina* DS2psM2a2 show a rumpled sheet-like morphology in cross-section through one of the fungal cell’s extracellular substances, whereas Mn oxides produced by *Stagonospora* sp. SRC11sM3a show a thread-like morphology [1,53]. We inferred that different fungal cell surface structures or EPS content may influence crystal morphology, which is frequently observed in other biomineralization processes [54,55]. 

The XRD pattern of the fungal Mn oxides shows poor crystallinity (Figure 5A), which is a typical mineralogical characteristic for both bacterial and fungal Mn oxides according to previous studies [2,36,39,42,43,56,57,58]. A previous study by Sasaki et al. [42] showed that the fungal manganese formed by *Paraconipthyrium* sp. was poorly crystalline birnessite. However, Miyata et al. [36] observed that the XRD patterns of manganese oxides produced by four ascomycete fungi correspond to the typical poorly crystalline vernadite (δ-MnO_2_). This finding aligns with Grangeon et al.’s report, which indicated that three ascomycete fungi (*Acremonium* sp. strain KR21-2 and unclassified *Pleosporales* strains IRB20-1 and IRB20-20, isolated from manganese coatings on stream pebbles, also produced vernadite [58]. *Acremonium strictum* KR21-2 exhibited a todorokite structure [39,59], while *Acremonium strictum* DS1bioAY4a generated δ-MnO_2_ in a liquid medium, but when it grew on the solid medium, it produced both δ-MnO_2_ and todorokite [1]. 

In contrast to fungal Mn oxides, the bacterial Mn oxides produced by *L. discophora* SP-6, *P. putida* MnB1, and *Bacillus* sp. SG-1 closely resemble δ-MnO_2_ or birnessite [60,61,62], which are layered-type Mn oxides containing a large number of structural defects and water, explaining why they are abundant in aquatic environments and can oxidize metal ions quickly [59]. In contrast, fungi in drier environments oxidize metal ions more slowly, and defect-free tunnel-type Mn oxides are often produced by manganese-oxidizing fungi [59]. However, our result shows that MnF107 produces both layered-type birnessite and tunnel-type pyrolusite and ramsdellite [3,44]. SAED failed to distinguish those MnO_2_ polymorphs in that it showed their diffusive rings (inset of Figure 5B). However, we indeed observed a different lattice spacing of Mn oxides corresponding to the (1 1 $\bar{2}$) and (1 1 1) planes of birnessite (0.215 nm and 0.225 nm), the (3 0 1) plane of ramsdellite (0.255 nm), and the (1 1 0) plane of pyrolusite (0.310 nm). This suggests that the fungal Mn oxides are not only poorly crystalline but also have a polycrystalline structure.

The XPS and K-edge XANES spectra have often been used to determine the oxidation state of manganese. In this study, we used the XPS spectra to identify the oxidation state of Mn in the fungal Mn oxides. The Mn 2p_1/2_ peak is centered at 653.9 eV, and the Mn 2p_3/2_ peak occurs at 642.2 eV, with a spin-energy separation of 11.7 eV, which is in good agreement with the previous report for a MnO_2_ phase [46,63]. The satellite peak at 647 eV is associated with the Mn 2p_1/2_ peak at 652.4 eV and the Mn 2p_3/2_ peak at 640.7 eV, indicating the presence of un-oxidized Mn(II) in the fungal Mn oxides [45,46], which is confirmed by the Mn3s spectrum. The spin-energy separations of 4.8 eV and 5.9 eV are in agreement with those expected for Mn(IV) and Mn(II) [64,65,66], indicating an incomplete conversion of Mn(II) to Mn(IV). However, we did not detect that the intermediate Mn(III) was present in the fungal oxides. Mn(III) is thermodynamically unstable and may be disproportionate to the yields of Mn(II) and Mn(IV) [67,68]. Miyata et al. [36,43] used the K-edge XANES spectra to characterize the chemical state of manganese in fungal Mn oxides. Their results showed that the fungal Mn oxides consisted predominantly of Mn(IV), usually with some extent of Mn(II) also present, indicating the conversion of Mn(II) to Mn(IV). This result is consistent with our observation of the chemical state of manganese in the fungal Mn oxides by XPS spectra.

## 4. Materials and Methods

### 4.1. Isolation and Identification of Manganese-Oxidizing Fungus

The marine sediment was collected from the estuary of Xinhe river in Beidaihe, China (N 119°31.2′18.893″ and E 39°50.2′11.903″). Five grams of the sample was suspended in 200 mL of autoclaved seawater and mixed thoroughly. Then, the resulting supernatant was spread on the AY agar media [1] and supplemented with a MnSO_4_ (CAS No. 10034-96-5, Merck, Rahway, NJ, USA) solution to a final concentration at 1 mM. The agar plates were incubated at 28 °C for 2 weeks. The dark brown fungal colonies were selected as the candidates for manganese-oxidizing fungi. 

The fungal genomic DNA was extracted, and PCR (polymerase chain reaction) amplification of the fungal internal transcribed spacer (ITS) commenced with the primer pair ITS1/ITS4 using the method described by Wang et al. [69]. The PCR product was purified and sequenced on an Applied Biosystems 3730 DNA Analyzer (Thermo Fisher Scientific, Waltham, MA, USA). The obtained sequence was employed to identify the fungus using the BLASTn program against the NCBI database. The ITS sequences with their relatives were aligned using the Clustal W algorithm in the MEGA 10 software, and phylogenetic trees were constructed using the maximum likelihood method.

### 4.2. Light and Fluorescence Microscopy

The fungus was inoculated on the AY agar plate covered with a cellophane membrane, facilitating the removal of the fungus from the plate for light and fluorescence microscopic observations. For fluorescence microscopic (Olympus BX51, Tokyo, Japan) observations, the AY media were supplemented with 0.5 μg/L DAPI before pouring the plates. 

### 4.3. Scanning Electronic Microscopy (SEM) and Energy-Dispersive X-ray Spectroscopy (EDS)

The fungal cultures were incubated at 28 °C for 14 d on a shaker in liquid AY media with or without 1 mM MnSO_4_. The fungal mycelial balls were collected and washed in deionized water. After dehydration, the desiccated mycelia were mounted on stubs and sputter-coated with Au/Pd film (10 nm) using a Gatan 682 sputter coater. The samples were investigated under scanning electron microscopy (SEM) (FEI Quanta 200F, Thermo Fisher Scientific, US) equipped with an energy-dispersive X-ray spectroscope (EDS, EDAX Genesis 2000, EDAX Inc., Pleasanton, CA, USA), which was operated at an accelerating voltage of 15 kV. The images were acquired in backscatter electron (BSE) mode.

### 4.4. Transmission Electron Microscopy (TEM)

Mycelial balls grown in liquid AY media were freeze-dried and pre-fixed in 2.5% (*w*/*v*) glutaraldehyde in 0.1 M sodium phosphate buffer (pH 7.2) for 2 h at room temperature, followed by thoroughly washing with phosphate buffer. They were then post-fixed in 1% aqueous osmium tetroxide at room temperature for 2 h. Each of the fixed samples was dehydrated in a gradient ethanol solution for 10 min from 30% to 100%. The samples were embedded with LR White resin and incubated at 60 °C overnight, and then thin sections of 60 nm thickness were prepared using a LeicaLeicaUC7. Thin sections were stained with uranyl acetate for 20 min and with lead citrate for 5 min, then mounted on 100-mesh grids and examined under a JEOL JEM-1400 transmission electron microscope.

### 4.5. X-ray Diffraction (XRD)

The Mn(II)-oxidizing fungus was inoculated on the top surface of the cellophane membrane spread on the AY agar plates and incubated at 28 °C for 14 d. The grown mycelia with Mn oxides were scraped off and washed with deionized water, then homogenized with ethanol and vacuum-dried. The collected dry solid samples were ground into powder and back-packed into a quartz holder. The XRD diffraction patterns were recorded using Cu-kα radiation at 40 kV and 35 mA with scanning of 10° to 80° 2θ at a scanning speed of 0.05°/s.

### 4.6. High-Resolution Transmission Electron Microscopy (HRTEM) and Selected Area Diffraction (SAED)

The fungal Mn oxides were suspended in water. A drop of aqueous sample was loaded on a carbon-coated copper grid, which was allowed to dry for an hour. The HRTEM micrograph images and SAED pattern were recorded on a TEM-JEOL 2100 F instrument operated at an accelerating voltage of 200 kV.

### 4.7. X-ray Photoelectron Spectroscopy (XPS)

The oxidation state of fungal Mn oxides was determined with X-ray photoelectron spectroscopy (XPS) using a Thermo ESCALAB 250Xi spectrometer. The instrument was operated at 150 W under a vacuum of 1 × 10^−10^ mbar using monochromatic Al-kα radiation (1486.6 eV). The binding energies were calibrated to the C 1s peak at 284.8 eV. The wide scans and the narrow scans were recorded using a 650 μm spot size, whereas fixed-pass energies of 100 eV and 20 eV were used for survey wide scans and narrow scans, respectively. The collected spectra include a survey scan and the Mn 2p and Mn 3s regions.

## 5. Conclusions

The results presented here demonstrate that the strain *Neoroussoella solani* MnF107 can oxidize manganese (II). Mn oxides are initially formed on the fungal cell wall and then aggregated as particles around the fungal hyphal surface. The Mn oxides have a filamentous texture and are formed in the position of EPS enveloping the fungal cells. The fungal Mn oxides are structurally composed of birnessite (δ-MnO_2_), pyrolusite (β-MnO_2_), and ramsdellite (γ-MnO_2_). The oxidation state of fungal Mn oxides is confirmed as predominantly Mn(IV), indicating the conversion of Mn(II) to Mn(IV).

## Data Availability

Data are contained within the article.
